# Dietary Supplementation of Crossbred Pigs with Glycerol, Vitamin C, and Niacinamide Alters the Composition of Gut Flora and Gut Flora-Derived Metabolites

**DOI:** 10.3390/ani14152198

**Published:** 2024-07-28

**Authors:** Panting Wei, Wenchen Sun, Shaobin Hao, Linglan Deng, Wanjie Zou, Huadong Wu, Wei Lu, Yuyong He

**Affiliations:** 1 Jiangxi Province Key Laboratory of Animal Nutrition and Feed, Engineering Research Center of Feed Development, Jiangxi Agricultural University, Nanchang 330045, China; 18679954991@163.com (P.W.); sun1221_wc@163.com (W.S.); haoshaobin1775@163.com (S.H.); denglinglan2021@163.com (L.D.); zou19970812@163.com (W.Z.); lw20030508@163.com (W.L.); 2 College of Animal Science and Technology, Jiangxi Agricultural University, Nanchang 330045, China; whd0618@163.com

**Keywords:** glycerin, vitamins, meat color, intestinal microbiota, metabolites, finishing pig, feces

## Abstract

**Simple Summary:**

The redness of *longissimus dorsi* can be improved significantly by administrating growing–finishing pigs with glycerin, vitamin C, and niacinamide; however, it remains unclear if these supplements can have significant impacts on the compositions of gut microbiota and metabolites. The results indicated that dietary supplementation with glycerin, vitamin C, and niacinamide significantly promoted the growth of iron-acquiring microbiota in feces, reduced the expression of some virulence factor genes of fecal pathogens, and increased the fecal levels of ferric ion, L-proline and some secondary bile acids. The findings of this study provide new ideas for improving meat quality by modulating gut flora and metabolites with feed interventions.

**Abstract:**

The addition of glycerin, vitamin C, and niacinamide to pig diets increased the redness of *longissimus dorsi*; however, it remains unclear how these supplements affect gut microbiota and metabolites. A total of 84 piglets (20.35 ± 2.14 kg) were randomly allotted to groups A (control), B (glycerin-supplemented), C (vitamin C and niacinamide-supplemented), and D (glycerin, vitamin C and niacinamide-supplemented) during a feeding experiment. Metagenomic and metabolomic technologies were used to analyze the fecal compositions of bile acids, metabolites, and microbiota. The results showed that compared to pigs in group A, pigs in group D had lower virulence factor expressions of lipopolysaccharide (*p* < 0.05), fatty acid resistance system (*p* < 0.05), and capsule (*p* < 0.01); higher fecal levels of ferric ion (*p* < 0.05), allolithocholic acid (*p* < 0.01), deoxycholic acid (*p* < 0.05), tauroursodeoxycholic acid dihydrate (*p* < 0.01), glycodeoxycholic acid (*p* < 0.05), L-proline (*p* < 0.01) and calcitriol (*p* < 0.01); and higher (*p* < 0.05) abundances of iron-acquiring microbiota (*Methanobrevibacter*, *Clostridium*, *Clostridiaceae*, *Clostridium_sp_CAG_1000*, *Faecalibacterium_sp_CAG_74_58_120*, *Eubacteriales_Family_XIII_Incertae_Sedis*, *Alistipes_sp_CAG_435*, *Alistipes_sp_CAG_514* and *Methanobrevibacter_sp_YE315*). Supplementation with glycerin, vitamin C, and niacinamide to pigs significantly promoted the growth of iron-acquiring microbiota in feces, reduced the expression of some virulence factor genes of fecal pathogens, and increased the fecal levels of ferric ion, L-proline, and some secondary bile acids. The administration of glycerol, vitamin C, and niacinamide to pigs may serve as an effective measure for muscle redness improvement by altering the compositions of fecal microbiota and metabolites.

## 1. Introduction

Nutrients exert vital effects on the health, performance, and meat quality of animals by regulating intestinal microbiota and metabolites [1,2]. Glycerol is a byproduct of biodiesel production from plants [3]. It is used in animal production as a promising feed supplement because optimum glycerol supplementation decreases feed costs and improves growth and meat quality in animals [4,5]. In addition, glycerol is a precursor of reuterin, produced by a few strains of *Lactobacillus* sp., and reuterin can inhibit the adhesion of pathogenic bacteria in the gut [6]. Feeding low-protein diets supplemented with niacinamide at 360 mg/kg to pigs increased nitrogen retention and decreased the content of intramuscular fat in the longissimus dorsi muscle [7]. Vitamin C supplementation is beneficial in increasing muscle redness in goats [8]. At high doses, vitamin C provides optimal protection against protein damage such as for the myoglobin of muscle fibers [9], with a significant increase in the endurance performance of undamaged type I and II muscle fibers [10].

The supplementation of glycerol, vitamin C, or niacinamide to animals can also affect the composition of gut microbiota. Glycerol addition to the feed of broiler chicken decreased the abundance of *Enterobacteriaceae* and increased butyrate production in cecum [11]. Dietary glycerol supplementation to seabass also increased the abundances of *Burkholderia* and *Vibrio* and the bacterial diversity [12]. Vitamin C administration reduced the abundance of Bacteroidetes but elevated the abundance of spirochetes in the gut of Wistar rats [13]; niacinamide (nicotinamide) modulated the growth of bacteria such as *Odoribacter*, *Flexispira,* and *Bifidobacterium* in the gut of mice [14]. However, the synergistic effect of glycerol, vitamin C, and niacinamide on gut microbiota has not been reported.

It is reported that the gut microbiome and microbial metabolites exert a considerable effect on the meat quality of farm animals, because they often act as substrates or signaling factors in influencing meat quality [15]. Nutritional interventions targeting the intestinal microbiota exhibit excellent potential in improving meat quality by fostering myofiber conversion, promoting intramuscular fat deposition, or changing amino acid composition [15,16]. Our previous study demonstrated that dietary supplementation of crossbred pigs with glycerol, vitamin C, and niacinamide decreases the incidence of pale, soft, and exudative muscle by increasing the redness value and percentage of type I myofibers in the longissimus dorsi muscle [17]. However, it remains unclear whether their co-supplementation can reshape the metabolite profile and microbial composition in pig feces. It is well known that metagenomics and metabolomics are currently the most popular tools for analyzing the composition and metabolism of the gut microbiome, because metagenomics can be used to identify marker microbes at the species level and metabolomics is used to find out the differential metabolites [16]. Herein, we used multiomics to identify the differential microbiota and metabolites in the feces of growing–finishing pigs supplemented with glycerol, vitamin C, and niacinamide.

## 2. Materials and Methods

### 2.1. Experimental Animals

Eighty-four weaned piglets (Duroc × Large White × Landrace) with an average initial body weight of 20.35 ± 2.14 kg were randomly assigned to groups A, B, C, or D. Each group contained 21 weaned piglets having free access to feed (Table 1) and water throughout the 103-day feeding trial [17].

### 2.2. Sample Collection

Thus, 21 pigs in each group were raised in three pens, and each pen had 7 pigs. After the adaptation period, fecal samples were collected at day 23, 38, 53, 68, 83, and 98. At each timepoint of collection, three pigs in each pen were randomly selected and used for the collection of fecal samples from the anus of pigs; then, the samples from three pens in the same group were mixed, sub-sampled, placed in 10 mL sterile plastic tubes, and stored in liquid nitrogen for determining iron and glycerol concentrations and characterizing gut microbiota and metabolites. 

### 2.3. Iron and Glycerol Contents in Fecal Samples

A ferrozine-based colorimetric assay was performed to quantify ferric and ferrous iron levels in the fecal samples [18], and glycerol concentrations in the fecal samples were determined using ELISA kits (Shanghai Enzyme-linked Biotechnology Co. Ltd., Shanghai, China) following the manufacturer’s protocols.

### 2.4. Metagenomic Sequencing

The T Guide S96 Magnetic Soil/Stool DNA kit (Tiangen Biotech, Beijing, China) was used to extract the genomic DNA from pig feces. The concentration and purity of extracted DNA were measured with the Nanodrop 2000 (Thermo Fisher, Wilmington, MA, USA). The qualified DNA was digested into fragments, barcoded with adapters (adpter3=′′AGATCGGAAGAGCACACGTCTGAACTCCAGTCAC′′;adpter5=′′AGATCGGAAGAGCGTCGTGTAGGGAAAGAGTGT′′) and purified. The purified DNA fragments with adapters were amplified using VAHTSTM Universal Plus DNA Library Prep Kits (ND617-02, Vazyme Biotech Co., Ltd., Nanjing, China) in Eppendorf 6333 DNA thermocycler (Eppendorf, Netheler-hinz, Hamburg, Germany) under the following cycling conditions: initial denaturation at 95 °C for 3 min, followed by eight cycles at 98, 60, and 72 °C for 20, 15, and 30 s, respectively, and a final extension at 72 °C for 5 min. The amplified products were purified using Vazyme DNA clean beads. The purified products were qualified with Qsep 400 Bio-Fragment Analyzer (BiOptic Bio-Tech, Nanjing, China) and quantified with Qubit 3.0 Fluorometer (Invitrogen, Eugene, OR, USA) to generate amplicon libraries. The libraries were pooled in an equimolar ratio and sequenced at Biomarker Technologies Co., Ltd. (Beijing, China) on an Illumina NovaSeq 6000 platform (Illumina, San Diego, CA, USA). Raw metagenomic data were deposited in the Sequence Read Archive of the National Center for Biotechnology Information under the BioProject ID PRJNA1055599.

Sequences of low quality were removed from raw reads using fastp software to generate clean reads with high quality [19]. The clean reads were used for metagenome assembly using MEGAHIT software (version 1.2.2, https://github.com/voutcn/megahit) [20], and the assembly outputs were evaluated using QUAST [21]. The MetaGeneMark software (version 3.26, http://exon.gatech.edu/meta_gmhmmp.cgi) was used to predict coding genes [22], and the MMseqs2 software (version 11-e1a1c, https://mmseqs.com/) was used to construct a non-redundant gene set by removing redundancy with a similarity threshold at 95% and coverage threshold at 90% [23]. Functional gene annotation and functional and taxonomic diversity analyses were performed based on this gene set.

### 2.5. Metabolomic Examination of Feces Using Untargeted Liquid Chromatography/Mass Spectrometry (LC/MS)

First, a 50 mg fecal sample, 1000 μL of a mix of methanol, acetonitrile, and water (2:2:1, *v*/*v*/*v*), and 10.0 μL of internal standards (20 mg/L) were placed in each tube, vortexed for 30 s at 4.0 °C, then ground at 45 Hz for 10 min and extracted ultrasonically for 10 min. Second, the tubes were allowed to stand for an hour at −20 °C, centrifuged at 4.0 °C for 15 min at 12,000 rpm, then a total of 500 μL of the supernatant was taken into each Eppendorf (EP) tube, and dried in a vacuum freeze dryer. Subsequently, 160 μL mix of acetonitrile and water (2:2:1, *v*:*v*:*v*) was added into each EP tube, vortexed for 30 s, and extracted ultrasonically for 10 min again, then centrifuged at 4 °C and 12,000 rpm for 15 min. Finally, 120 μL of the supernatant was placed into a vial. Metabolomic analysis was performed using an LC/MS system (Waters Ltd., Milford, MA, USA) consisting of an ACQUITY UPLC I-Class PLUS System, ultra-high-performance liquid tandem Xevo G2-XS QTof, a high-resolution mass spectrometer, coupled with an ACQUITY UPLC HSS T3 column. The raw data were collected using the MassLynx V4.2 software. The Progenesis QI software (Waters Corporation, Massachusetts, United States) was used for the extraction, alignment, and identification of peaks using online METLIN database, and Biomark’s self-built library. The R package was used to perform orthogonal partial least squares discriminant analysis (OPLS-DA) modeling, and the model’s reliability was verified 200 times via permutation tests. The fold change (FC)-value > 1, *p*-value < 0.05, and variable importance in projection (VIP)-value > 1 were used to screen differential metabolites.

### 2.6. Analysis of Fecal Bile Acids Based on Targeted LC/MS

Fecal samples ground in liquid nitrogen were mixed with ultra-pure water (Milli-Q water; Massachusetts, USA) by vortexing to produce a diluent. Next, 100 μL of the diluent was mixed with 300 μL of internal standards containing a mix of methanol and acetonitrile (2:8, *v*/*v*). The mix was allowed to stand for 30 min on ice, then centrifuged at 12,000 rpm for 10 min at 4.0 °C. The supernatant was pipetted into vials for targeted LC/MS analysis. Bile acids in fecal samples were quantitated by Novogene Co., Ltd. (Beijing, China) with an ultra-high-performance system in which liquid chromatography is coupled to tandem mass spectrometry (UHPLC-MS/MS) (ExionLC AD UHPLC-QTRAP 6500+; AB SCIEX Corp., Boston, MA, USA). The Analyst 1.6.3 software (Sciex, Darmstadt, Germany) was used for data acquisition, and peak picking and calibration were performed using MultiQuant 3.0.2 software (Sciex, Germany). The FC-value > 1.2 and *p*-value < 0.05 were adopted to screen the differential bile acids.

### 2.7. Statistical Analysis

Statistical analyses were performed using the R package (version 3.6.1; R Foundation for Statistical Computing, Vienna, Austria). The non-parametric Wilcoxon test was used to test the differences between groups, and the non-parametric Kruskal–Wallis rank-sum test was used to test the significance of the differences among groups. Data are expressed as the mean ± standard error of the mean, and the statistical significance was set at *p* < 0.05.

## 3. Results

### 3.1. Concentrations of Glycerol and Ferric and Ferrous Irons in Fecal Samples

The concentrations of glycerol and ferric and ferrous irons in fecal samples are presented in Table 2. The results revealed no significant difference in the glycerol content of fecal samples among the treatment groups; however, dietary supplementation with glycerol, a mix of vitamin C and niacinamide, or a mix of glycerol, vitamin C, and niacinamide tended to decrease the levels of glycerol in feces. Pigs in group B exhibited lower (*p* < 0.05) concentrations of ferric ions in their fecal samples than those observed in the fecal samples of pigs in groups A and D. The concentrations of ferric and ferrous ions were higher (*p* < 0.05) in the fecal samples of pigs in group D than in the fecal samples of pigs in group B. Thus, the concentrations of fecal ferric ions were affected by the interaction between vitamin C and niacinamide or the interaction between glycerol and the complex of vitamin C and niacinamide (*p* < 0.01). The interaction between glycerol and the mix of vitamin C and niacinamide significantly impacted the fecal ferrous ions levels (*p* < 0.05).

### 3.2. Fecal Metagenomic Profiles

**Figure 1:**
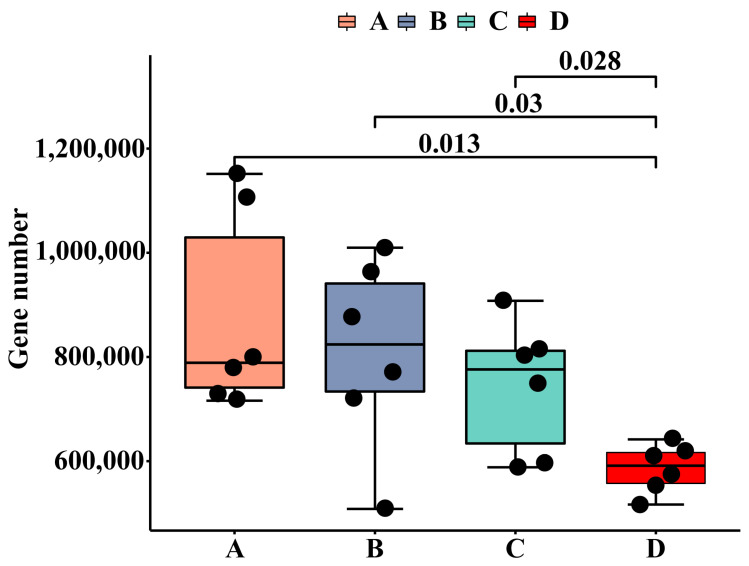
The box plot presented the difference of gene number between groups. *x*-axis: groups; *y*-axis: non-redundant gene number; the upper and lower end of the box: The upper and lower interquartile range (IQR); the median line: median; upper and lower edges: maximum and minimum inner bounding value (1.5-times IQR); points outside the upper and lower edges: outliers. The number on the line between the columns is the *p*-value.

**Figure 2:**
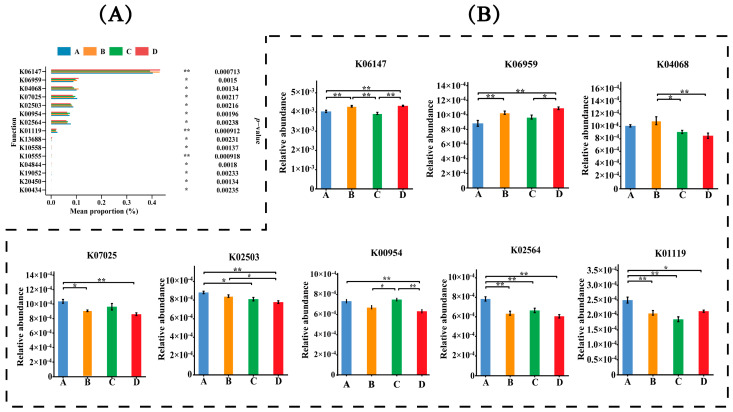
The comparisons of differential KO pathways. KO pathways with *p*-value smaller than 0.05 in Kruskal–Wallis rank-sum test were shown in the bar plots. (**A**): Bar plots show the relative abundance of the top 15 remarkably different KO pathways (*p* < 0.05). The abscissa shows the abundance of KO pathways, and the ordinate is the names of differential KO pathways. (**B**): Comparison of significant differences of differential KO pathways among groups A–D. The abscissa shows the names of treatment groups, and the ordinate is the relative abundance of KO pathways. Marks on the line between the columns indicate a statistical significance: * indicates *p* < 0.05; ** indicates *p* < 0.01.

**Figure 3:**
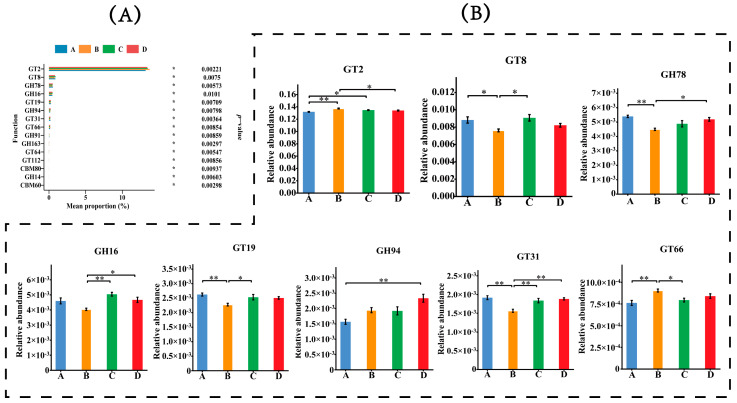
The comparisons of differential CAZymes family genes. Functional genes with p-value smaller than 0.05 in Kruskal–Wallis rank-sum test were shown in the bar plots. (**A**): Bar plots show the relative abundance of the top 15 remarkably different CAZy family genes (*p* < 0.05). The abscissa shows gene abundance, and the ordinate is the names of differential CAZymes genes. (**B**): Comparison of significant differences of differential CAZymes family genes among groups A–D. The abscissa shows the names of treatment groups, and the ordinate is the relative abundance of CAZymes family gene. Marks on the line between the columns indicate a statistical significance: * indicates *p* < 0.05; ** indicates *p* < 0.01.

**Figure 4:**
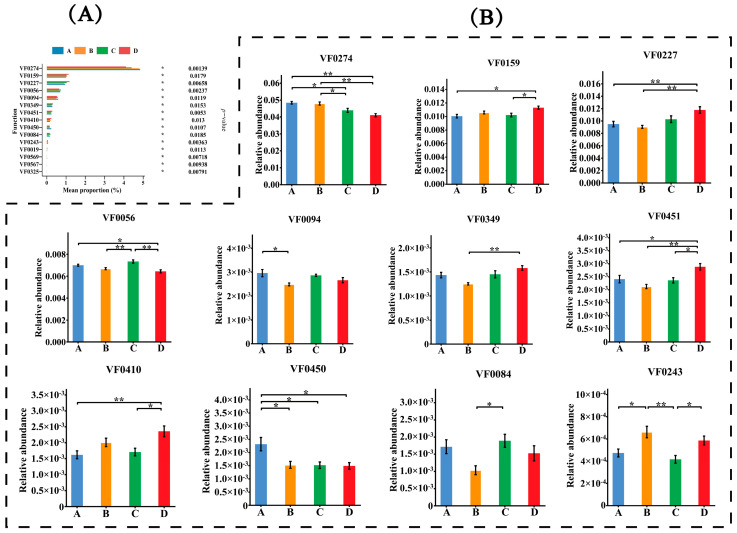
The comparisons of virulence factors. Virulence factors with *p*-value smaller than 0.05 in Kruskal–Wallis rank-sum test were shown in the bar plots. (**A**): Bar plots show the relative abundance of the top 15 remarkably different virulence factors (*p* < 0.05). The abscissa shows the abundance of virulence factors, and the ordinate is the names of differential virulence factors. (**B**): Comparison of significant differences of differential virulence factors among groups A–D. The abscissa shows the names of treatment groups, and the ordinate is the relative abundance of virulence factors. Marks on the line between the columns indicate a statistical significance: * indicates *p* < 0.05; ** indicates *p* < 0.01.

Each fecal sample had an average of 54,228,808 ± 2,757,465 clean reads, 667,655 ± 31,531 contigs, and 994,842 ± 51,033 genes after quality control, de novo assembly, and gene prediction. The differences in gene numbers between groups are presented in Figure 1. The non-redundant genes were annotated using the Kyoto Encyclopedia of Genes and Genomes (KEGG) database, the carbohydrate-active enzymes database (CAZy), and the virulence factor database (VFDB) to profile the KEGG orthology (KO) pathway, CAZymes, and virulence factors, respectively. The comparisons of gene functions are presented in Figure 2, Figure 3 and Figure 4. A total of 103,931 functional genes were annotated in the KO database. The genes were mainly enriched in the K06147 (ATP-binding cassette, subfamily B, bacterial), K06959 (testis-expressing protein, protein Tex), K04068 (anaerobic ribonucleoside-triphosphate reductase activating protein), K07025 (putative hydrolase of the haloacid dehalogenase superfamily), K02503 (histidine triad family protein), K00954 (pantetheine phosphate adenylyltransferase), K02564 (glucosamine-6-phosphate deaminase), and K01119 (2′,3′-cyclic-nucleotide 2′-phosphodiesterase/3′-nucleotidase) functional categories. Group D exhibited an upregulated (*p* < 0.01) expression of genes in the K06147 and K06959 functional categories but a downregulated expression of those in the K07025 (*p* < 0.01), K02503 (*p* < 0.01), K00954 (*p* < 0.01), K02564 (*p* < 0.01), and K01119 (*p* < 0.05) categories than that observed in groups A and C. The number of functional genes annotated in CAZy was 8454, which were mainly associated with glycosyltransferases (GTs) and glycoside hydrolases (GHs), including GT2, GT8, GH78, GH16, GT19, GH94, GT31, and GT66. Group D exhibited a higher (*p* < 0.01) proportion of GH94 than that observed in group A; group C exhibited a higher proportion of GT2 (*p* < 0.05) than that observed in group A and higher proportions of GT8 (P<0.05), GH16 (*p* < 0.01), GT19 (*p* < 0.05), and GT31 (*p* < 0.01) than those observed in group B. Group B exhibited higher proportions of GT2 (*p* < 0.01) and GT66 (*p* < 0.01) but lower proportions of GT8 (*p* < 0.05), GH78 (*p* < 0.01), GT19 (*p* < 0.01), and GT31 (*p* < 0.01) than those observed in group A. In addition, 5650 functional genes annotated in VFDB were mainly enriched in the VF0274 (capsule), VF0159 (heat shock protein 60, Hsp60), VF0227 (Escherichia coli heme uptake), VF0056 (lipopolysaccharide, LPS), VF0094 (pyoverdine), VF0349 (fibronectin-binding protein A, FbpA), VF0451 (multiple transferable resistance system), VF0410 (listeriolysin S), VF0450 (fatty acid resistance system, FarAB), VF0084 (Xcp type II secretion system), and VF0243 (fibronectin/fibrinogen-binding proteins, FBPs) categories. Group D exhibited downregulated expression of genes in the VF0274 (*p* < 0.01), VF0056 (*p* < 0.05), and VF0450 (*p* < 0.05) categories but upregulated expression of those in the VF0159 (*p* < 0.05), VF0227 (*p* < 0.01), VF0451 (*p* < 0.05), and VF0410 (*p* < 0.01) categories than in group A. Group C exhibited decreased expression of genes in the VF0274 (*p* < 0.05) and VF0450 (*p* < 0.05) categories than in group A. Group B exhibited increased expression of genes in the VF0094 (*p* < 0.05) and VF0450 (*p* < 0.05) categories but decreased expression of those in the VF0243 (*p* < 0.05) category than in group A.

**Figure 5:**
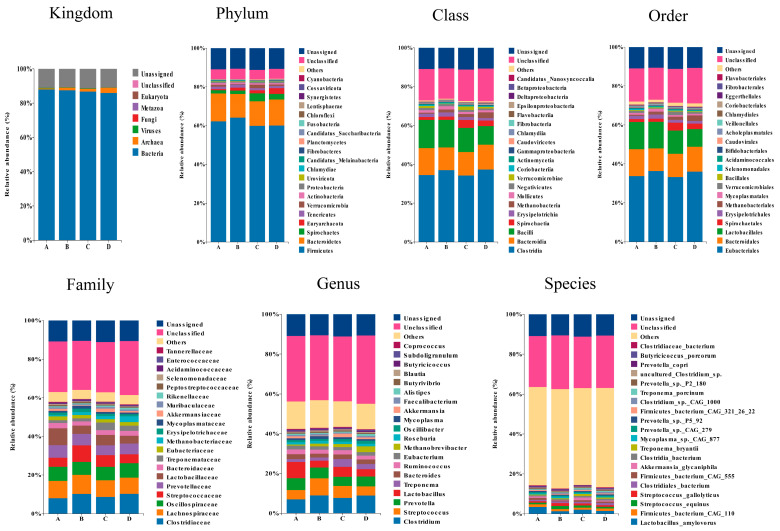
Microbial community composition displaying the top abundant taxa across all samples. The taxonomic composition of the fecal microbiome is shown at various taxonomic levels: Kingdom, Phylum, Class, Order, Family, Genus, and Species. Each bar represents the relative abundance of different microbial taxa in fecal samples from four treatment groups: A (control), B (glycerin-supplemented), C (vitamin C and niacinamide-supplemented), and D (glycerin, vitamin C, and niacinamide-supplemented). The color-coded bar plot shows the average bacterial distribution in each group.

**Figure 6:**
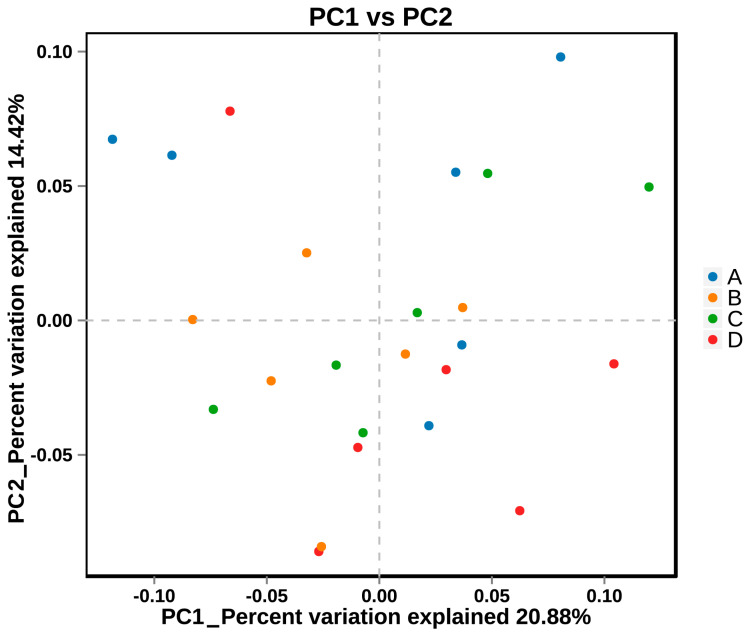
PCoA plot based on Bray–Curtis distance matrix of fecal microbial community structures of the four treatment groups. Treatment groups are differentiated with different colored circles: A (control), B (glycerin-supplemented), C (vitamin C and niacinamide-supplemented), and D (glycerin, vitamin C, and niacinamide-supplemented). The two axes of the plot explained 35.3% of the variance, with X and Y axes explaining 20.88% and 14.42% of the total variation, respectively.

**Figure 7:**
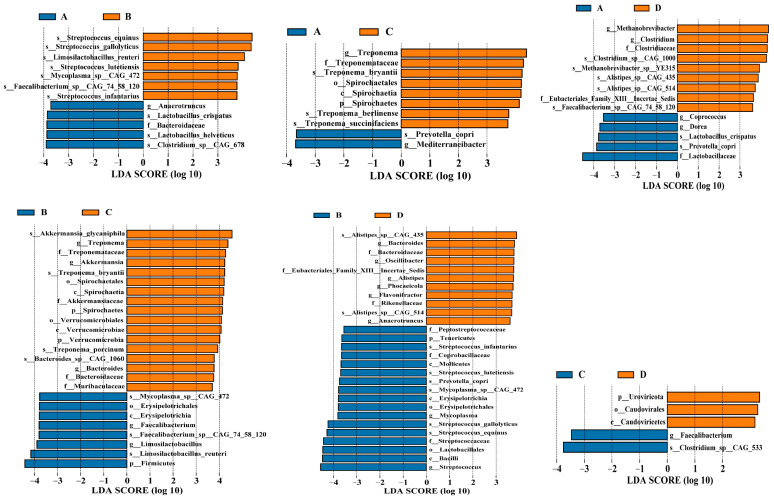
The differential microbiota between groups based on Linear Discriminant Analysis (LDA) Effect Size (LEfSe). This figure compares the relative abundance of specific microbial taxa among the treatment groups: (A) vs. (B), (A) vs. (C), (A) vs. (D), (B) vs. (C), (B) vs. (D), and (C) vs. (D). Each plot shows taxa with significant differences in abundance between groups, with the LDA score (log 10) indicating the effect size. Orange bars represent higher abundance in the first group, while blue bars represent higher abundance in the second group. Significant taxa include various bacterial species at different taxonomic levels, with notable differences highlighted for each comparison.

All non-redundant genes were annotated in the non-redundant protein database, and the taxonomic composition of the fecal microbiome at the kingdom, phylum, class, order, family, genus, and species levels is presented in Figure 5. In addition, the result of principal coordinates analysis (PCoA) for microbiome is shown in Figure 6. The results of alpha diversity are listed in Table 3, and the data indicated that group D had a lower Ace and Chao 1 index than group A (*p* < 0.05). Differential microbiota with significant differences between the groups were identified using the Wilcoxon rank-sum test and LEfSe analysis, and the results are shown in Figure 7. Compared with that in group A, group B exhibited higher (*p* < 0.05) relative abundance of *s_Streptococcus_equinus*, *s_Limosilactobacillus_reuteri*, *s_Streptococcus_gallolyticus*, *s_Streptococcus_lutetiensis*, *s_Mycoplasma_sp._CAG_472*, *s_Streptococcus_infantarius*, and *s_Faecalibacterium_sp._CAG_74_58_120* and lower (*p* < 0.05) relative abundance of *g_Anaerotruncus*, *s_Lactobacillus_crispatus*, *f_Bacteroidaceae*, *s_Lactobacillus_helveticus*, and *s_Clostridium_sp._CAG_678*; group C exhibited higher (*p* < 0.05) relative abundances of *g_Treponema*, *f_Treponemataceae*, *o_Spirochaetales*, *p_Spirochaetes*, *s_Treponema_bryantii*, *c_Spirochaetia*, *s_Treponema_berlinense*, and *s_Treponema_succinifaciens* and lower (*p* < 0.05) relative abundance of *s_Prevotella_copri* and *g_Mediterraneibacter*; and group D exhibited higher (*p* < 0.05) relative abundance of *g_Methanobrevibacter*, *g_Clostridium*, *s_Clostridium_sp._CAG_1000*, *s_Methanobrevibacter_sp._YE315*, *s_Alistipes_sp._CAG_435*, *f_Clostridiaceae*, *s_Alistipes_sp._CAG_514*, *f_Eubacteriales_Family_XIII_Incertae_Sedis*, and *s_Faecalibacterium_sp._CAG_74_58_120* and lower (*p* < 0.05) relative abundance of *g_Coprococcus*, *f_Lactobacillaceae*, *s_Lactobacillus_crispatus*, *s_Prevotella_copri*, and *g_Dorea*.

Pigs in group D exhibited significantly higher concentrations of ferric and ferrous ions in fecal samples than those in group A. Therefore, the iron uptake-related components of differential microbiota in fecal samples between groups D and A were identified using a non-redundant protein database, and the results are listed in Table 4. The data indicated that pigs in group D had nine different microbiota that absorbed iron with more iron uptake-related components; however, pigs in group A had three different microbiota that enriched iron, with few iron uptake-related components.

### 3.3. Fecal Metabolomic Profiles

**Figure 8:**
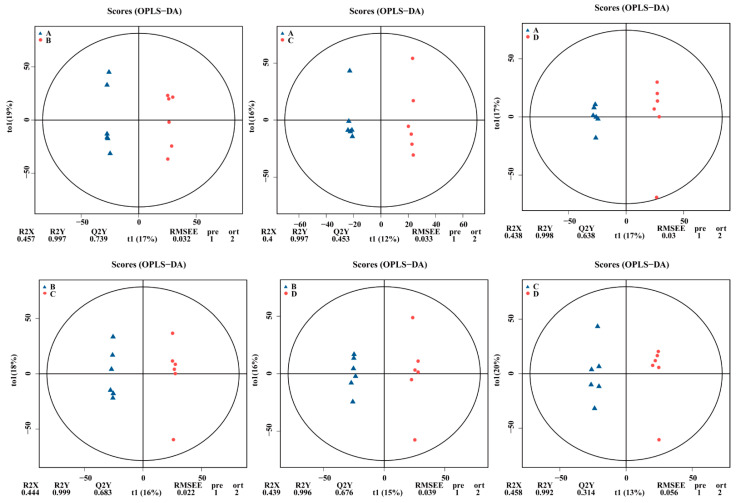
The OPLS-DA score plot of primary metabolites obtained from fecal samples analyzed by untargeted liquid chromatography/mass spectrometry. It compares the OPLS-DA score of A vs. B, A vs. C, A vs. D, B vs. C, B vs. D, and C vs. D. The *x*-axis (t1) represents the predicted component (inter group difference component), and the *y*-axis (to1) represents the orthogonal component (intra group difference component). R2Y represents the percentage of all sample variables explained by the model, Q2Y represents the percentage of all sample variables predicted by the model.

**Figure 9:**
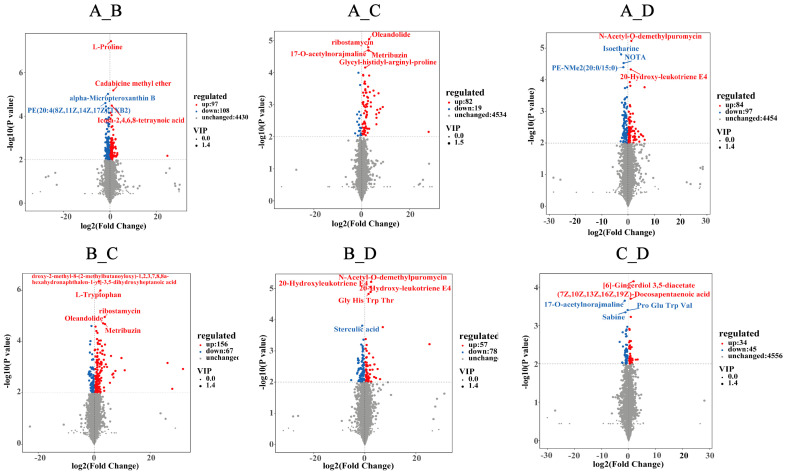
The Volcano plots of differential metabolites between groups. Each dot in the volcano plots represents a metabolite, and the blue ones represent the significantly down-regulated metabolites, the red dots represent the significantly up-regulated metabolites, the gray dots represent the metabolites that have no significant difference between the two groups. The ordinate shows the −log10 (*p*-value), and the abscissa is the log2 (fold change) value. The ions with VIP >  1.0 and *p*  <  0.05 were considered to be important differential metabolites.

**Figure 10:**
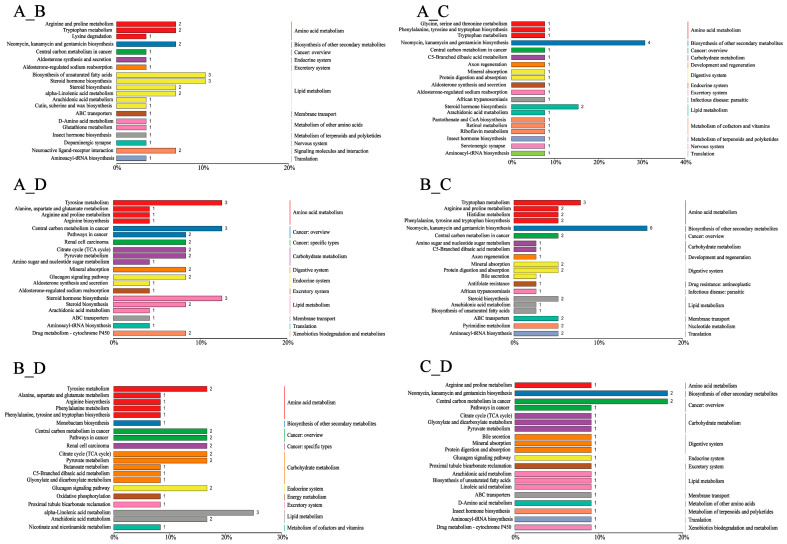
The top 20 terms KO pathway enrichment bar plot of differential metabolites. The abscissa represents enrichment radio, and the ordinate represents enrichment pathways. The different colored entries represent the hierarchical classification annotations of the KEGG pathway, corresponding to KO pathway level 3 and KEGG pathway names. The length of the column represents the number of differential metabolites annotated by the pathway.

We identified 4635 metabolites matching against unique standards, and the metabolite profiles between the groups were separated using OPLS-DA (Figure 8). Moreover, 924 differential metabolites presented in Figure 9 were annotated in the KEGG database. The top 20 terms of the KO pathway level 3 are shown in Figure 10. The ABC transporter and mineral absorption pathways are related to iron absorption, and the differential metabolites annotated in these pathways were L-proline, L-tryptophan, and calcitriol. The results presented in Table 5 show that groups B, C, and D exhibited higher (*p* < 0.01) levels of L-proline than those in group A in the ABC transporter pathways. Moreover, L-proline was upregulated (*p* < 0.01) between groups A and B and between groups C and D; L-proline and L-tryptophan were upregulated (*p* < 0.01) between groups A and C and between groups B and C, respectively; and group A exhibited lower calcitriol levels than in group D (*p* < 0.01) in the mineral absorption pathways.

### 3.4. Concentrations of Fecal Bile Acids

The differential levels of bile acids in the fecal samples of pigs in groups A–D are listed in Table 6. The levels of DCA, GCA, and GDCA were higher (*p* < 0.01), but those of α-MCA were lower (*p* < 0.05) in the fecal samples of pigs in group B than in the fecal samples of those in group A. The concentrations of DCA (*p* < 0.05) and TUDCA (*p* < 0.01) were higher, but those of HCA (*p* < 0.05) were lower in the fecal samples of pigs in group C than in the fecal samples of those in group A. The levels of alloLCA and TUDCA (*p* < 0.01) and DCA and GDCA (*p* < 0.05) were higher in the fecal samples of pigs in group D than in those of pigs in group A. Pigs in group B exhibited lower levels of alloLCA (*p* < 0.05) and TUDCA (*p* < 0.01) in their fecal samples than in those of pigs in group C. The alloLCA and TUDCA contents were lower (*p* < 0.01) in the fecal samples of pigs in group B than in those of pigs in group D. The levels of TUDCA (*p* < 0.05) were higher in the fecal samples of pigs in group C than in those of pigs in group D.

## 4. Discussion

The addition of glycerol can boost the growth of *Bacillus* spp. and *Microbacterium* spp. that uptake iron from the environment by producing siderophores [24,25]. Moreover, glycerol can be metabolized to produce acetate, butyrate, lactate, succinate, and ethanol by promoting the growth of gut microbes, e.g., *Streptococcus*, *Faecalibacterium*, L. *reuteri*, *Lachnospiraceae*, and *Lachnoclostridium* [26,27,28]. The oral administration of vitamin C to healthy humans at 1000 mg/day improves the relative abundance of *Lachnospiraceae* (*p* < 0.05) and reduces those of *Bacteroidetes* and *Enterococci* (*p* < 0.01) and *Gemmiger formicilis* (*p* < 0.05) [29]. Another study reported that oral vitamin C supplementation has impact on the abundance of Bacteroidetes in the gut of patients with non-alcoholic fatty liver [30]. Mice receiving niacinamide at 250 mg/kg BW had an impact on gut microbiota [14]. Our results show that pigs fed a diet containing glycerol, vitamin C, and niacinamide exhibited a significantly higher abundance of short-chain fatty acid-producing bacteria, e.g., *Faecalibacterium_sp._CAG_74_58_120* and iron-acquiring bacteria, including *Clostridium*, *Methanobrevibacter*, *Methanobrevibacter_sp._YE315*, *Clostridiaceae*, *Clostridium_sp._CAG:1000*, *Alistipes_sp._CAG_435*, *Alistipes_sp._CAG_514*, and *Faecalibacterium_sp._CAG:74_58_120*, compared with that in pigs not fed glycerol, vitamin C, and niacinamide. This could explain the higher concentrations of iron (*p* < 0.05) and acetate (*p* < 0.01) and lower levels of glycerol (*p* < 0.05) in fecal samples of pigs fed diets supplemented with glycerol, vitamin C, and niacinamide. Previous studies have reported that iron-dependent bacteria (*E. coli* and *Clostridium* spp.) increase fecal iron by decreasing iron absorption [31], whereas iron-independent bacteria (*Lactobacilli* sp.) decrease fecal iron by increasing iron absorption [32].

High retention of fecal iron can decrease the risk of ferroptosis in muscles by decreasing the iron uptake from the gut lumen because muscle ferroptosis can decrease muscle redness by increasing metmyoglobin content or damaging the normal function of muscle cells. Additionally, the high relative abundance of *P. copri* induces obesity and inflammation [33], intensifies *Listeria monocytogenes* infection, and increases intestinal permeability; however, supplementation with glycerol, vitamin C, and niacinamide significantly decreases the relative abundance of *P. copri*.

Supplementation with glycerol, vitamin C, and niacinamide also altered the composition of gut microbial metabolites; pigs fed these dietary supplements had higher fecal levels of L-proline than those fed non-supplemented diets. L-proline can inhibit ferroptosis by eliminating lipid hydroperoxides with CoQH2 produced by proline dehydrogenase and dihydroorotate dehydrogenase and augmenting reduced glutathione biosynthesis by shifting the redox stress signaling threshold with NRF2 and the antiporter system xc− [34,35]. Pigs fed diets supplemented with glycerol, vitamin C, and niacinamide had a higher relative abundance of potential pathogens in fecal samples than those who did not receive these supplements. *Methanobrevibacter* sp. and *Methanobrevibacter_sp._YE315* are methane-producing microbes; methane is one of the causes of constipation and enteritis [36]. However, clinically, fewer gut disease symptoms were observed in pigs fed glycerol, vitamin C, and niacinamide, which may be related to the improved host resistance against pathogens by L-proline [37]. Gut pathogens exert negative effects on host health through the production of LPS or lipooligosaccharides, adhesins, capsules, siderophores, iron-scavenging systems, and toxins [38]. Nutrients such as iron critically impact the virulence and colonization of pathogens [39]. Moreover, certain virulence factors are regulated by iron [40]. Dietary supplementation with glycerol, vitamin C, and niacinamide significantly increased fecal iron levels, which significantly inhibited the expression of the capsule, LPS, and fatty acid resistance system genes but promoted the expression of Hsp60 genes, multiple transferable resistance system genes, and listeriolysin S genes and *E. coli* heme uptake. Downregulation of the capsule, LPS, and fatty acid resistance systems contributed to host health by reducing the growth, toxicity, and adhesion of gut pathogens [41,42]. Hsp60 can protect intestinal epithelial cells from inflammation [43]. Bacteriocins, e.g., listeriolysin S, help evade excessive inflammation by reducing the growth of intestinal pathogens [44]. Listeriolysin S has been identified in *Streptococcus pyogenes*, *Staphylococcus aureus*, and *Clostridium botulinum* [45,46]. Clayton et al. (2014) reported that listeriolysin S was detected in several non-pathogenic *Listeria innocua* strains [47]. It can reduce the growth of certain Gram-positive bacteria, including *Lactococcus lactis* and *S. aureus* [48].

Bile acids are cholesterol metabolites that emulsify dietary lipids and regulate bile acid biosynthesis and immune signaling [49]. The pool of intestinal bile acids consists of primary bile acids synthesized by hepatocytes and secondary bile acids produced by the gut bacterial metabolism [50]. The results of this study indicate that pigs fed diets supplemented with glycerol, vitamin C, and niacinamide had a higher abundance of *Clostridium* sp. and higher levels of DCA and LCA than pigs fed non-supplemented diets. Some *Clostridium* spp. can convert primary bile acids to DCA and LCA [50], which, in turn, can directly inhibit the germination and outgrowth of *Clostridioides difficile* [51,52]. *Clostridium scindens* and other *Clostridium* spp. can inhibit *C. difficile* by secreting antimicrobials, which are more effective in protecting against *C. difficile* infection when used together with DCA and LCA [53].

The KO annotation results showed that microbial genes were mainly enriched in K06147 and K06959. Previous studies have found that K06147 (ATP-binding cassette, subfamily B, bacteria) can translocate drugs, lipids, peptides, and iron [54] and positively regulate the expression of hyaluronidase genes [55]. Hyaluronidase can break down extracellular hyaluronic acid to provide carbon and energy for bacterial growth, including pathogens [56]. The deposition and remodeling of hyaluronic acid precede inflammation, and an increase in hyaluronidase levels may alleviate inflammation by accelerating hyaluronic acid decomposition [57]. The testis-expressing protein K06959 (protein Tex) is a highly conserved bacterial protein that functions in fatty acid synthesis and exerts antibacterial activity by regulating lysosome function [58]. Pigs fed glycerol–vitamin C–niacinamide-supplemented diets exhibited higher proportions of K06147 and K06959 than those in the control group, implying that supplementation with glycerol, vitamin C, and niacinamide together contribute toward improving gut health in pigs.

The CAZyme analysis revealed that the four treatment groups significantly affected the proportions of GT2, GT8, GH78, GH16, GT19, GH94, GT31, and GT66. The GTs participate in the biosynthesis of functional saccharides, whereas GHs are implicated in glycosidic bond hydrolysis [59]. The fecal samples of pigs fed a diet containing glycerol or a complex of vitamin C and niacinamide exhibited higher proportions of GT2 enzymes than those of pigs not receiving glycerol, vitamin C, or niacinamide. The GT2 enzyme is a type of synthase for the production of bacterial cellulose and hyaluronic acid and has a vital role in the synthesis of bacterial cellulose, which can coat bacterial and fungal cells to form cell aggregates, capsules, or biofilms [60,61]. Thus, the microorganisms in the guts of pigs fed a diet containing glycerol or a complex of vitamin C and niacinamide may exhibit stronger biofilm-forming abilities than those in pigs not receiving glycerol, vitamin C, and niacinamide. The GH94 enzyme can degrade dietary fiber constituents and carbohydrates by catalyzing the phosphorolysis of glycans into sugar 1-phosphates and glycan chains [62]. Pigs fed a diet containing glycerol, vitamin C, and niacinamide exhibited the highest proportions of GH94, indicating an enhanced capability to digest complex carbohydrates.

## 5. Conclusions

Supplementation with glycerol, vitamin C, and niacinamide alters the composition of microbiota and metabolites in pig feces. Pigs with better muscle redness and higher type I myofiber percentage had a higher abundance of iron-acquiring microbiota, higher levels of iron, L-proline, DCA, and LCA, and lower expression of the virulence factor genes of pathogens in their feces. These findings provide novel insights into obtaining the desired types of muscle fibers and myoglobin by targeting the gut microbiota and related metabolites through nutrient administration.

## Figures and Tables

**Table 1:** Diet composition and nutrient level (%, on dry matter basis).

	Group A		Group B		Group C		Group D	
	10–60 kg	60–120 kg	10–60 kg	60–120 kg	10–60 kg	60–120 kg	10–60 kg	60–120 kg
Ingredients								
Corn	59.00	64.00	48.00	52.00	58.89	63.89	47.89	51.89
Wheat bran	16.00	16.00	15.00	15.00	16.00	16.00	15.00	15.00
Soybean meal	14.00	10.00	15.00	11.00	14.00	10.00	15.00	11.00
Fishmeal	2.00	0.00	2.00	0.00	2.00	0.00	2.00	0.00
Rapeseed meal	5.00	6.00	6.00	8.00	5.00	6.00	6.00	8.00
4% Premix ^(1)^	4.00	4.00	4.00	4.00	4.00	4.00	4.00	4.00
Glycerin	0.00	0.00	10.00	10.00	0.00	0.00	10.00	10.00
Vitamin C	0.00	0.00	0.00	0.00	0.06	0.06	0.06	0.06
Niacinamide	0.00	0.00	0.00	0.00	0.05	0.05	0.05	0.05
Total	100.00	100.00	100.00	100.00	100.00	100.00	100.00	100.00
Nutrient levels ^(2)^								
Metabolizable energy (MJ/kg)	12.15	12.24	12.26	12.31	12.15	12.24	12.26	12.31
Crude protein	17.30	15.10	17.34	15.18	17.22	15.05	17.36	15.21
Crude fiber	3.84	3.68	3.69	3.78	3.74	3.71	3.47	3.63
Ether extract	3.81	3.68	3.43	3.50	3.94	3.88	3.57	3.65
Calcium	0.79	0.65	0.82	0.70	0.73	0.69	0.78	0.63
Total phosphorus	0.67	0.54	0.63	0.55	0.69	0.59	0.66	0.60
Lysine	1.09	0.84	1.09	0.86	1.09	0.84	1.09	0.86
Methionine + Cystine	0.57	0.44	0.56	0.43	0.57	0.44	0.56	0.43

^(1)^ The premix provided the following per kg of the diet in 10 to 60 kg stage: VA 2750 IU, VD3 500 IU, VE 11 IU, VK3 0.50 mg, VB1 0.55 mg, VB2 1.55 mg, VB6 0.80 mg, VB12 10 µg, Niacotinic acid 7.5 mg, pantothenic acid 6.5 mg, biotin 0.08 mg, Cu (CuSO_4_•5H_2_O) 5 mg, Zn (ZnSO_4_•1H_2_O) 17.5 mg, Mn (MnSO_4_•1H_2_O) 5 mg, Fe (FeSO_4_•1H_2_O) 21.3 mg, Se (Na_2_SeO_3_) 0.07 mg, I (KI) 0.08 mg, Co(CoCl_2_•1H_2_O) 0.06 mg, Lysine 90 g, Methionine 71 g. The premix provided the following per kg of the diet in 60 to 90 kg stage: lys 90 g, VA 1600 IU, VD3 300 IU, VE 11 IU, VK3 0.50 mg, VB1 0.48 mg, VB2 1.10 mg, VB6 0.65 mg, VB12 9 µg, Niacotinic acid 6.5 mg, pantothenic acid 6.0 mg, biotin 0.06 mg, Cu (CuSO_4_•5H_2_O) 5 mg, Zn (ZnSO_4_•1H_2_O) 13.5 mg, Mn (MnSO_4_•1H_2_O) 5 mg, Fe (FeSO_4_•1H_2_O) 17.0 mg, Se (Na_2_SeO_3_) 0.07 mg, I (KI) 0.08 mg, Co(CoCl_2_•1H_2_O) 0.06 mg, Lysine 69 g, Methionine 49 g. ^(2)^ Levels of metabolizable energy, lysine, methionine and cystine are calculated values. Levels of crude protein, crude fiber, ether extract, calcium and total phosphorus are measured values.

**Table 2:** Levels of glycerol and ferrous and ferric ions in fecal samples.

	Group A	Group B	Group C	Group D	SEM	*p*-Value		
						GLY	VC + NAM	GLY × (VC + NAM)
Glycerol (µg/g)	122.02	117.16	111.22	100.29	19.70	0.325	0.092	0.702
Ferric ion (µg/g)	43.35 ^b^	32.04 ^c^	37.84 ^bc^	54.30 ^a^	2.15	0.363	0.007	<0.001
Ferrous ion (µg/g)	12.69 ^ab^	10.45 ^b^	11.41 ^ab^	15.27 ^a^	3.40	0.524	0.173	0.024

SEM, standard error of the mean; GLY, glycerol; VC, vitamin C; NAM, niacinamide; group A, without addition of glycerin, vitamin C and niacinamide; group B, addition of 10% glycerin, 0% vitamin C and 0% niacinamide; group C, addition of 0% glycerin, 0.06% vitamin C and 0.05% niacinamide; group D, addition of 10% glycerin, 0.06% vitamin C and 0.05% niacinamide. Means within a row followed by different lowercase letters differ significantly (*p* < 0.05). *p*-value means the effect of GLY, (VC + NM) or the interaction of GLY and (VC + NM) on the parameter within the same row.

**Table 3:** Alpha diversity of fecal microbiota in different groups.

	Group A	Group B	Group C	Group D	SEM	*p*-Value
Ace	9612.02 ^a^	9072.30 ^ab^	9048.48 ^ab^	8129.06 ^b^	896.70	0.024
Chao 1	9584.16 ^a^	9046.86 ^ab^	9025.50 ^ab^	8121.57 ^b^	891.96	0.027
Shannon	6.42	6.53	6.45	6.47	0.12	0.467
Simpson	0.99	0.99	0.99	0.99	0.00	0.152

Group A was without the addition of glycerin, vitamin C, and niacinamide; group B featured the addition of 10% glycerin, 0% vitamin C, and 0% niacinamide; group C featured the addition of 0% glycerin, 0.06% vitamin C, and 0.05% niacinamide; group D featured the addition of 10% glycerin, 0.06% vitamin C, and 0.05% niacinamide. Means within a row followed by different lowercase letters differ significantly (*p* < 0.05).

**Table 4:** Iron uptake-related components of differential microbiota in fecal samples between group D and group A.

	Differential Microbiota	Iron Uptake-Related Components of Differential Microbiota
Group D	*s_Methanobrevibacter_sp._YE315*	ABC transporter permease
	*g_Methanobrevibacter*	Siderophore transport system ATP-binding protein YusV
		Ferredoxin oxidoreductase
		Ferrous iron transport protein B
		Desulfoferrodoxin
		ABC transporter permease
		ABC transporter ATP-binding protein
		Iron ABC transporter substrate-binding protein
		Iron ABC transporter permease
		Iron ABC transporter permease
	*g_Clostridium*	Siderophore/surfactin synthetase-related protein
		Siderophore
		Siderophore transport system permease protein yfhA
		Siderophore transport system ATP-binding protein YusV
		Pyruvate:ferredoxin (flavodoxin) oxidoreductase
		Ferredoxin
		4Fe-4S ferredoxin
		ABC transporter ATP-binding protein
		ABC transporter permease
		Ferrous iron transporter
	*f_Clostridiaceae*	Pyruvate ferredoxin oxidoreductase
		Ferredoxin
		ABC transporter ATP-binding protein
		ABC transporter permease
		ABC transporter substrate-binding protein
	*s_Clostridium sp. CAG:1000*	ABC transporter ATP-binding protein
		ABC-type transport system involved in Fe-S cluster assembly permease component
		ABC transporter-related protein
		ABC transporter permease protein
	*s_Methanobrevibacter_sp._YE315*	ABC transporter permease
	*s_Alistipes_sp._CAG_435*	Ferritin
		2-oxoacid:ferredoxin oxidoreductase γ-subunit
		ABC transporter ATP-binding protein
		Efflux ABC transporter permease protein
	*s_Alistipes_sp._CAG_514*	Ferredoxin
		Ferredoxin-NADP^+^ reductase subunit-α
		ABC transporter ATP-binding protein
	*s_Faecalibacterium_sp. CAG:74_58_120*	ABC transporter ATP-binding protein
		ABC transporter permease
		ABC transporter substrate-binding protein
Group A	*s_Prevotella_copri*	Iron ABC transporter permease
		ABC transporter substrate-binding protein
		ABC transporter permease
	*g_Dorea*	Metal ABC transporter permease
		ABC transporter ATP-binding protein
	*g_Coprococcus*	Ferredoxin
		ABC transporter ATP-binding protein
		ABC transporter permease

ABC, ATP-binding cassette. Group A, without addition of glycerin, vitamin C and niacinamide; group D, addition of 10% glycerin, 0.06% vitamin C and 0.05% niacinamide.

**Table 5:** Changes in the levels of differential metabolites between groups in the ATP-binding cassette (ABC) transporter and mineral absorption pathways.

#ID	Name	Groups	Fold Change (FC)	log_2_FC	*p*-Value	VIP
ABC transporters (ko02010)						
pos_2704	l-Proline	B/A	1.3386	0.4208	<0.0000	2.3914
neg_1618	l-Proline	C/A	2.4404	1.2871	0.0038	2.2233
pos_2704	l-Proline	D/A	1.4592	0.5451	0.0037	2.0634
neg_1618	l-Proline	C/B	6.2859	2.6521	0.0007	2.2128
pos_2704	l-Proline	D/C	1.3624	0.4461	0.0065	2.0721
Mineral absorption (ko04978)						
pos_2704	l-Proline	B/A	1.3386	0.4208	<0.0000	2.3914
neg_1618	l-Proline	C/A	2.4404	1.2871	0.0038	2.2233
neg_1957	l-Tryptophan	C/A	2.7506	1.4597	0.0004	2.5711
neg_3312	Calcitriol	D/A	0.3881	−1.3658	0.0053	1.8416
neg_1957	l-Tryptophan	C/B	4.6449	2.2156	<0.0000	2.3925
neg_1618	l-Proline	C/B	6.2859	2.6521	0.0007	2.2128
pos_2704	l-Proline	D/C	1.3624	0.4461	0.0065	2.0721

Group A, without addition of glycerin, vitamin C and niacinamide; group B, addition of 10% glycerin, 0% vitamin C and 0% niacinamide; group C, addition of 0% glycerin, 0.06% vitamin C and 0.05% niacinamide; group D, addition of 10% glycerin, 0.06% vitamin C and 0.05% niacinamide.

**Table 6:** Comparison of the levels of differential bile acids in fecal samples of different groups.

Name	Category	Concentration in Feces (ng/g)		Fold Change	*p*-Value
		Group B	Group A		
DCA	Secondary bile acid	445.87 ± 78.89	94.42 ± 30.18	4.72	0.0019
GCA	Primary bile acid	6.78 ± 0.90	2.59 ± 0.62	2.62	0.0032
α-MCA	Primary bile acid	84.50 ± 16.45	137.31 ± 15.81	0.62	0.0431
GDCA	Secondary bile acid	4.53 ± 0.40	2.03 ± 0.36	2.25	0.0008
		Group C	Group A		
DCA	Secondary bile acid	442.21 ± 94.41	94.42 ± 30.18	4.68	0.0127
TUDCA	Secondary bile acid	49.10 ± 3.13	15.65 ± 1.53	3.14	<0.0000
HCA	Primary bile acid	335.59 ± 31.15	646.12 ± 95.80	0.52	0.0214
		Group D	Group A		
alloLCA	Secondary bile acid	6455.71 ± 509.98	4193.75 ± 335.40	1.54	0.0041
DCA	Secondary bile acid	811.36 ± 188.50	94.42 ± 30.18	8.59	0.0121
TUDCA	Secondary bile acid	37.25 ± 3.28	15.65 ± 1.53	2.38	0.0001
GDCA	Secondary bile acid	6.52 ± 1.41	2.03 ± 0.36	3.21	0.0235
		Group B	Group C		
alloLCA	Secondary bile acid	3670.87 ± 316.31	5505.35 ± 504.44	0.67	0.0116
TUDCA	Secondary bile acid	15.29 ± 1.99	49.10 ± 3.13	0.31	<0.0000
		Group B	Group D		
alloLCA	Secondary bile acid	3670.87 ± 316.31	6455.71 ± 509.98	0.57	0.0009
TUDCA	Secondary bile acid	15.29 ± 1.99	37.25 ± 3.28	0.41	0.0002
		Group C	Group D		
TUDCA	Secondary bile acid	49.10 ± 3.13	37.25 ± 3.28	1.32	0.0259

DCA, deoxycholic acid; GCA, glycocholic acid; α-MCA, α-muricholic acid; GDCA, glycodeoxycholic acid; TUDCA, tauroursodeoxycholic acid dihydrate; HCA, hyocholic acid; and alloLCA, allolithocholic acid; group A, without addition of glycerin, vitamin C and niacinamide; group B, addition of 10% glycerin, 0% vitamin C and 0% niacinamide; group C, addition of 0% glycerin, 0.06% vitamin C and 0.05% niacinamide; group D, addition of 10% glycerin, 0.06% vitamin C and 0.05% niacinamide.

## Data Availability

The original contributions presented in the study are included in the article, and further inquiries can be directed to the corresponding author.
